# Supplementary material for: Surface Microbiome Profiling of Dental Elevators Using Third‐Generation Sequencing: Implications for Infection Control in Dental Practice
Source: Microbiologyopen. 2025 Dec 8;14(6):e70178. doi: 10.1002/mbo3.70178 (PMC12685768; doi:10.1002/mbo3.70178)
Supplement: Supplementary file 1 — customer_backup. [file MBO3-14-e70178-s001.zip › customer_backup/customer_backup/function_analysis/BugBase/treat/otu_contributions/order/treat.order.Contains_Mobile_Elements.pdf]

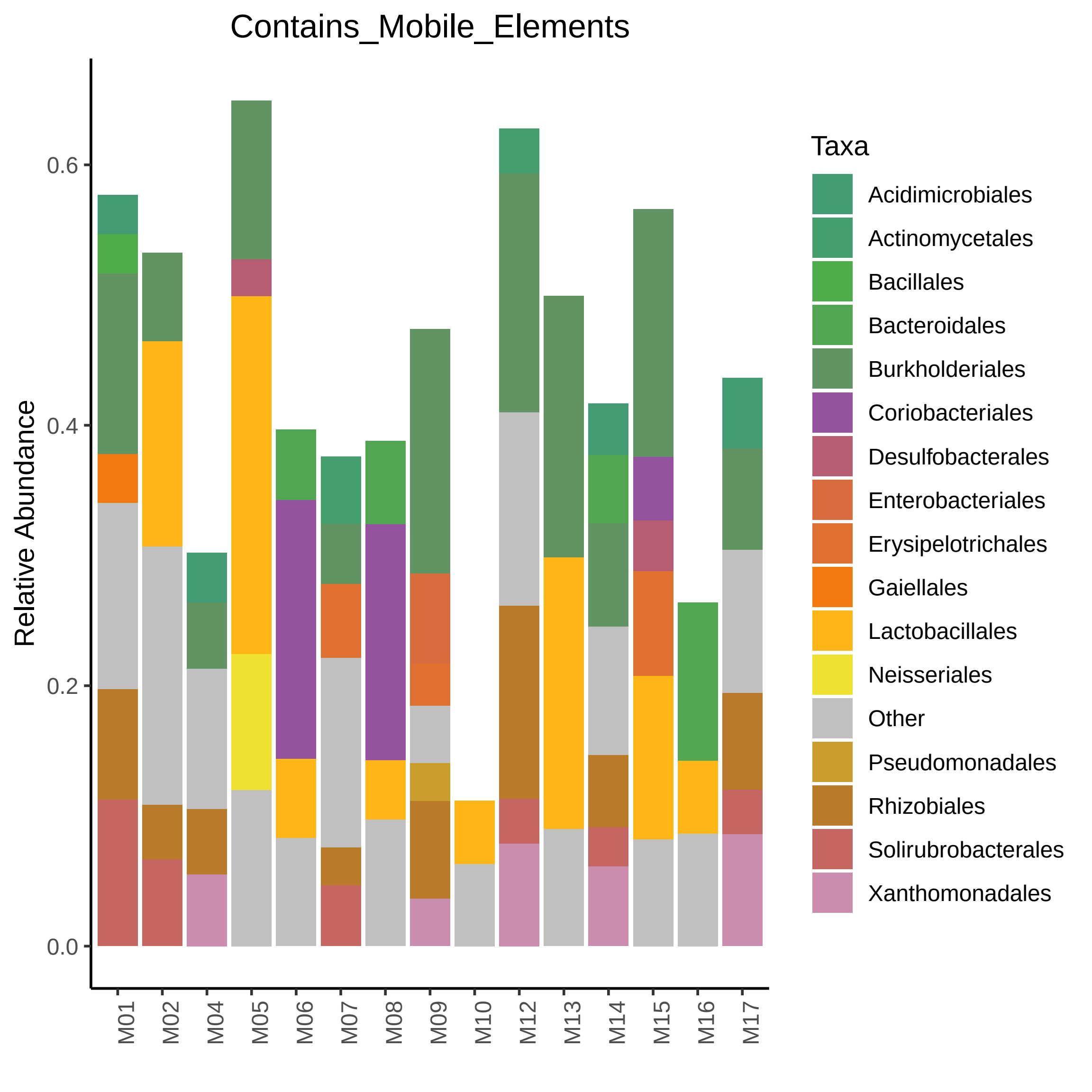

Contains_Mobile_Elements
Relative Abundance
0.0
0.2
0.4
0.6
M01
M02
M04
M05
M06
M07
M08
M09
M10
M12
M13
M14
M15
M16
M17
Taxa
Acidimicrobiales
Actinomycetales
Bacillales
Bacteroidales
Burkholderiales
Coriobacteriales
Desulfobacterales
Enterobacteriales
Erysipelotrichales
Gaiellales
Lactobacillales
Neisseriales
Other
Pseudomonadales
Rhizobiales
Solirubrobacterales
Xanthomonadales
